# Genome and Plasmid Sequences of *Escherichia coli* KV7, an Extended-Spectrum β-Lactamase Isolate Derived from Feces of a Healthy Pig

**DOI:** 10.1128/genomeA.00595-17

**Published:** 2017-09-21

**Authors:** Michael D. Bateman, Stefan P. W. de Vries, Srishti Gupta, Luca Guardabassi, Lina M. Cavaco, Andrew J. Grant, Mark A. Holmes

**Affiliations:** a Department of Veterinary Medicine, University of Cambridge, Cambridge, United Kingdom; b Ross University School of Veterinary Medicine, Basseterre, St. Kitts, West Indies; c National Food Institute, Technical University of Denmark, Kongens Lyngby, Denmark

## Abstract

We present single-contig assemblies for *Escherichia coli* strain KV7 (serotype O27, phylogenetic group D) and its six plasmids, isolated from a healthy pig, as determined by PacBio RS II and Illumina MiSeq sequencing. The chromosome of 4,997,475 bp and G+C content of 50.75% harbored 4,540 protein-encoding genes.

## GENOME ANNOUNCEMENT

A 2007 study (1) of healthy Danish pigs under prophylactic ceftiofur treatment uncovered a number of distinct strains of *Escherichia coli* carrying the *bla*_CTX-M-1_ gene and suggested that CTX-M-1 resistance spreads horizontally rather than clonally. A spontaneous nalidixic acid-resistant derivative of one of these strains that produces extended-spectrum β-lactamase (serotype 27, phylogenetic group D), named KV7, was investigated in a further study (2), showing that such strains thrive in pigs under treatment with several β-lactams. Sequencing of this isolate was performed in order to provide more detailed genetic data of a strain used for experimental infections of pigs.

The genomic and plasmid DNA was isolated from early exponential-phase cultures (LB medium) with Genomic-tip 100/G columns (Qiagen) and the plasmid midikit (Qiagen), respectively. Sequencing was performed using Illumina MiSeq and Pacific Biosciences technologies. Prior to PacBio RS II sequencing, the DNA was cleaned with a phenol extraction, and each preparation was sequenced on a single single-molecule real-time (SMRT) cell (Earlham Institute, Norwich, United Kingdom). For Illumina sequencing, DNA was sheared in an M220-focused ultrasonicator (Covaris). Sequencing libraries were prepared using the NEBNext Ultra II DNA library prep kit (New England Biolabs). Sequencing was performed using 250-bp paired-end sequencing on the MiSeq platform (V3 chemistry).

Assembly of genomic and plasmid DNA PacBio sequence reads was carried out at the Earlham Institute using the Hierarchical Genome Assembly Process (HGAP.3). Combining results from PacBio assemblies yielded single contigs for the chromosome and for five plasmids. A sixth plasmid was identified manually using a combination of raw reads and assembly fragments from both PacBio and Illumina sequencing assemblies; assembly of the Illumina reads was done with plasmidSPAdes version 3.9.0 (3, 4). This plasmid (P6) harbored a large repetitive region spanning approximately 2 kb. Polishing at the single-nucleotide polymorphism level was performed using Illumina reads with Snippy version 3.1. We did not find evidence of further plasmids.

The strain has multilocus sequence types (MLST) ST57 and ST533 using the original and enhanced *E. coli* MLST schemes, respectively. The KV7 chromosome was 4,997,475 bp with a GC content of 50.75%. The plasmid sizes were 223,014 bp (P1), 111,092 bp (P2), 71,546 bp (P3), 56,562 bp (P4), 32,688 bp (P5), and 39,510 bp (P6). Automated annotation was obtained with Prokka version 1.11 at Galaxy Queensland (5), revealing the presence of 4,540 chromosomal protein-encoding genes. In addition to *bla*_CTX-M-1_, a variety of antimicrobial resistance genes were identified on both the chromosome and several of the plasmids using BLAST together with the ARG-ANNOT database (6). These included genes predicted to encode resistance to aminoglycosides (*strA*, *strB*, and *aadA*), β-lactams (*bla*_*CTX-M-1*_, *bla*_*TEM-1D*_, *ampC1*, *ampC2*, and *ampH* and genes encoding penicillin-binding proteins), chloramphenicol (*catA1* and *cmlA*), a macrolide (*mphA*), sulfonamides (*sulI* and *sulII*), tetracycline (*tetB*), and trimethoprim (*dfrA*).

### Accession number(s).

The complete genome sequences of *E. coli* KV7 and plasmids have been deposited in the European Nucleotide Archive (ENA) (http://www.ebi.ac.uk/ena) and GenBank under accession no. LT795502 for the KV7 genome and LT795503, LT795504, LT795505, LT795506, LT795507, and LT795508 for the plasmids (study no. PRJEB19461).
